# Attenuating the Variability of Lipids Is Beneficial for the Hypertension Management to Reduce the Cardiovascular Morbidity and Mortality in Older Adults

**DOI:** 10.3389/fcvm.2021.692773

**Published:** 2021-06-17

**Authors:** Yuanli Dong, Xukui Liu, Yingxin Zhao, Qiang Chai, Hua Zhang, Yumei Gao, Zhendong Liu

**Affiliations:** ^1^Department of Community, Lanshan District People Hospital, Linyi, China; ^2^Basic Medical College, Shandong First Medical University, Jinan, China; ^3^Cardio-Cerebrovascular Control and Research Center, Institute of Clinical Medicine, Shandong Provincial Hospital Affiliated to Shandong First Medical University, Jinan, China; ^4^Department of Cardiology, Hekou District People Hospital, Dongying, China

**Keywords:** lipids variability, hypertension management, mortality, morbidity, statins

## Abstract

**Objective:** To investigate the beneficial of attenuating the variability of lipids to the hypertension management in older adults.

**Methods:** Between April 2008 and November 2010, 1,244 hypertensive patients aged ≥60 years were recruited and randomized into placebo and rosuvastatin groups. Outcomes and inter-visit plasma lipids variability were assessed.

**Results:** Over an average follow-up of 83.5 months, the coefficients of variation (CVs) in total cholesterol (TCHO), triglycerides, high-density lipoprotein cholesterol (HDL-c), and low-density lipoprotein cholesterol (LDL-c) were significantly lower in the rosuvastatin group than the placebo group (*p* < 0.05). The risks of composite cardiovascular event, myocardial infarction, coronary revascularization, heart failure, total stroke, ischemic stroke, cardiovascular death, and all-cause death were significantly lower in the rosuvastatin group than the placebo group (all p < 0.05). The differences in the risks were significantly diminished after the CVs for TCHO, triglycerides, HDL-c, and LDL-c were separately included as confounders. One-SD of CVs for TCHO, triglycerides, HDL-c, and LDL-c increment were significantly associated with the risks of composite cardiovascular event, myocardial infarction, heart failure, total stroke, ischemic stroke, cardiovascular death, and all-cause death, respectively (all *p* < 0.05).

**Conclusions:** Rosuvastatin significantly attenuated the intra-visit variability in lipids and decreased the risk of cardiovascular mortality and morbidity. Controlling the variability of lipids is as important as antihypertensive treatment to reduce the cardiovascular morbidity and mortality in the management of older hypertensive patients.

**Clinical Trial Registration:** ChiCTR.org.cn, ChiCTR-IOR-17013557.

## Introduction

Hypertension, a highly prevalent chronic condition in the adult population in China and worldwide, especially among people 60 years and older, is the leading risk factor for stroke, myocardial infarction, and even death (1–3). Therefore, hypertension poses a high financial burden to the healthcare system.

As is known, antihypertensive treatment is the most important measurement of hypertension management. The association between antihypertensive treatment and cardiovascular mortality and morbidity is well-established. However, >50% of patients treated for hypertension do not achieve the desired treatment goals (4–6). Hypertension commonly coexists with other cardiovascular risk factors such as dyslipidaemia, and risk factors interact and overlap in the pathophysiology of cardiovascular diseases (7–9).

Excessive plasma lipids has been demonstrated to be associated with cardio- and cerebro-vascular diseases (10, 11). Statin therapy is an effective therapeutic strategy for lowering-lipid and is recommended for patients with cardiovascular disease in several major guidelines (12–17). However, in several randomized controlled trials of statins, residual risk of cardiovascular diseases remains (18–20). In fact, the lipid level is fluctuating due to the changing of various factors such as medication adherence, season, diet style, and mood (21–25). Is the effect of variability in lipids as important as the level of lipids on cardio- and cerebro-vascular diseases? The association between statin treatment and lipids variability, and the effect of attenuating the variability of lipids on cardiovascular morbidity and mortality in the hypertension management in older adults are still unclear. In the present study, our main goal was to investigate this association and effect in older hypertensive adults.

## Methods

### Study Design and Patients

The data in this study were collected from a randomized, double-blind, placebo-controlled clinical trial (26). Between April 2008 and November 2010, 1,244 hypertensive patients aged 60 years and older were enrolled from community dwellings in six cities in Shandong, China. Hypertension was defined by systolic blood pressure (SBP) ≥ 140 mmHg and/or diastolic blood pressure (DBP) ≥ 90 mmHg, or treatment for hypertension. Patients were excluded if they met any of the following criteria: secondary hypertension; severe cardio- and cerebro-vascular diseases such as myocardial infarction and stroke in the previous 6 months; definite hypersensitivity or contraindication to statins, sartans, and thiazide diuretic; concurrent treatment with a statin or fibrate; concurrent treatment with a sartan, angiotensin converting enzyme inhibitor, or thiazide diuretic; chronic liver disease; chronic renal dysfunction; inflammatory muscle disease, such as polymyositis, or a creatine kinase level 3-fold higher than the normal upper limit; connective tissue diseases and malignancy; substantial psychiatric illness such as Alzheimer′s disease, Parkinson′s disease, schizophrenia, or seizures; plans to move or emigrate within 6 years; inability to walk to the clinic; and unwillingness to provide informed consent.

This study complied with the Declaration of Helsinki, adhered to good clinical practice guidelines, and was approved by the Research Ethics Committee of the Shandong Academy of Medical Sciences. Written informed consent was obtained from each participant. This trial is retrospectively registered with ChiCTR.org.cn, number ChiCTR-IOR-17013557.

### Randomization, Masking, and Intervention

After a 2-week washout period, patients were simultaneously and double-blindly randomized to antihypertensive intervention (1:1, placebo vs. telmisartan) or lipid moderating intervention (1:1, placebo *vs*. rosuvastatin). Telmisartan was provided at 40 mg with an increase to 80 mg once daily if needed, and hydrochlorothiazide was provided at 12.5 mg with an increase to 25 mg daily if needed. Hydrochlorothiazide was administered as an open-label medication and used as a background medication in the study. Rosuvastatin was given at 10 mg once daily. Computer-generated randomization was used according to the order of recruitment with a block size of eight, with stratification. The randomization was executed, and medications were supplied, by individuals who were not participants in this study. All investigators and patients were masked to treatment assignment during the double-blind phase. Except in cases of emergency, treatment allocations were not unmasked until the study was completed and after final clinical database lockdown.

In this study, we classified patients administered rosuvastatin placebo into the placebo group and patients administered rosuvastatin activator into the rosuvastatin group.

### Follow-Up

In the 2-week washout period, clinical follow-up visits were conducted weekly. After randomization, follow-up visits were conducted in months one, three, and six, and every 6 months thereafter until the conclusion of the trial. Demographic and clinical characteristics including age, sex, smoking status, alcohol consumption, and body mass index were recorded in each follow-up visit, and the data collected at the end of washout period were used as baseline data for further analysis. Investigators provided good medical care to patients independently of intervention assignment in the washout and follow-up periods. Blood pressure (BP) was measured with an automatic digital BP monitor (HEM-701, Omron Co., Ltd., Dalian, China). Plasma lipids and fasting plasma glucose (FPG) were detected with standard methods at baseline and in every annual follow-up visit. Concomitant use of open-label statins and/or sartans was not allowed. Medication compliance was evaluated by counting of the number of tablets taken.

### Assessment of Plasma Lipid Variability

Blood serum was collected in the morning after overnight fasting and stored at −80°C until analysis. Serum lipids including total cholesterol (TCHO), triglyceride (TG), high-density lipoprotein cholesterol (HDL-c), and low-density lipoprotein cholesterol (LDL-c) were measured at the central laboratory at the Basic Medical College & Institute of Basic Medicine, Shandong First Medical University & Shandong Academy of Medical Sciences. The inter-visit variability in serum lipids was calculated as the means of the intra-individual standard deviation (SD) and coefficient of variation (CV) over each individual's measurements with the use of post-baseline measurements (11). CV was defined as the SD corrected for the intra-individual mean lipid level over the same measurement period (11). The measurements of lipids at 6-month follow-up visit were used as the first measurements during the calculation to avoid bias caused by the onset of rosuvastatin administration.

### Outcomes

The primary outcome was the time until the first occurrence of any component of the composite endpoint: stroke, myocardial infarction, coronary revascularization, admission due to heart failure, or composite cardiovascular death. The secondary outcomes included all-cause mortality and newly incident diabetes mellitus. Stroke was defined as a neurological deficit lasting longer >24 h, with relevant findings of cerebral ischemic infarction, intracerebral hemorrhage, or subarachnoid hemorrhage in magnetic resonance imaging or computed tomography. Myocardial infarction was defined by symptoms of chest pressure and electrocardiograph changes, elevation in cardiac enzymes >2-fold above the normal upper limit, or coronary angiography and ventriculography. Heart failure was defined by typical clinical oedema with pulmonary congestion in chest roentgenogram, echocardiographic left ventricular dysfunction, and increased plasma brain natriuretic peptide levels. Newly incident diabetes mellitus was defined by FPG ≥7.0 mmol/L (≥126 mg/dL), post-challenge glucose ≥11.1 mmol/L (≥200 mg/dL), and/or glycated hemoglobin ≥6.5% (27). All reported outcomes were adjudicated by a blinded independent endpoint evaluation committee.

### Sample Size Determination and Power Calculation

The sample size was determined according to the incidence of stroke, because this incidence is ~5-fold higher than that of myocardial infarction in China (28, 29). The incidence of major vascular events in the rosuvastatin group was assumed to be 5.5% per year (30, 31). With a loss rate of <10%, a sample size of 1,200 patients was determined to be required. This study enrolled 1,244 patients and performed follow-up for an average of 6 years, thus achieving a power of 90% for detecting a 25% stroke reduction in patients randomized to the placebo group versus the rosuvastatin group (two-sided *p*-value < 0.05).

### Statistical Analysis

Data are presented as mean with SD, median with interquartile range (IQR; 25th and 75th percentiles), or numbers with percentages when appropriate. The normality of continuous data was determined with the Kolmogorov-Smirnov test. The differences were assessed with Student's *t*-test or Mann-Whitney *U*-test for continuous data and the chi-square test for categorical data between the placebo and rosuvastatin groups. We used a linear mixed model to assess the differences in the changes in BP, plasma lipid profiles, and FPG over time between the groups. Kaplan-Meier analysis with log-rank test was used to calculate cumulative event rates and compare times to outcomes. The Cox proportional hazards model with Wald test was used to assess the hazard ratio (HR) and 95% confidence interval (CI). In Cox hazard model 1, we did not adjust for any confounders. In model 2, the confounders included age, sex, smoking, alcohol consumption, education, baseline body mass index. In models 3, the confounders included mean in SBP, DBP, TCHO, TG, HDL-c, and LDL-c during the follow-up, as well as those included in model 2. To further investigate the associations between lipid variability and the outcomes, we used HRs expressing the risk associated with a one-SD increment in the CVs for lipids. Multiple sensitivity analyses were performed to assess the robustness of the findings: (1) adjustment for mean in SBP and DBP during the follow-up period, because BP level is closely associated with cardiovascular events (32, 33); (2) adjustment for mean FPG during the follow-up period; (3) adjustment for medication adherence (on the basis of pill count); and (4) use of the first diagnosed outcome during the trial period for analysis. All statistical analyses were performed in the SPSS for Windows software package, version 24.0 (SPSS Inc., Chicago, IL, USA). All tests of significance were two-sided, and a *p*-value < 0.05 was considered statistically significant.

## Results

### Patient Characteristics

The demographic and clinical characteristics at baseline and end of follow-up are shown in Table 1. The mean age was 70.11 years, and 47.5% were female. There were no significant differences in clinical characteristics including body mass index, BP, plasma lipids, and FPG between the placebo and rosuvastatin groups (all *p* > 0.05).

**Table 1:** Demographic and clinical characteristics at baseline and end of follow-up.

	**Baseline**			**End of follow-up**			*P-*value^*^	*P-*value^**^
	**Placebo group** **(*n* = 622)**	**Rosuvastatin group** **(*n* = 622)**	***P-*value**	**Placebo group** **(*n* = 552)**	**Rosuvastatin group** **(*n* = 581)**	***P-*value**		
Female [*n* (%)]	298 (47.9) 324	299 (48.1) 323	0.955	274 (49.6) 286	286 (49.2) 295	0.890	0.726	0.689
Age (years)	70.30 ± 6.18	69.92 ± 5.98	0.265	77.13 ± 6.21	77.05 ± 6.14	0.827	<0.001	<0.001
Smoking [*n* (%)]	163 (26.2) 459	151 (24.3) 471	0.486	115 (20.8) 437	116 (20.0) 465	0.717	0.031	0.072
Alcohol consumption [*n* (%)]	230 (37.0) 392	215 (34.6) 407	0.375	183 (33.2) 369	180 (31.0) 401	0.434	0.171	0.186
Education (years)	7.0 (4.0, 10.0)	7.0 (5.0, 10.0)	0.366	–	–	–	–	–
Body mass index (kg/m^2^)	24.63 ± 2.99	24.78 ± 3.25	0.410	24.46 ± 2.57	24.51 ± 3.03	0.765	0.299	0.137
SBP (mm Hg)	156.82 ± 9.90	156.41 ± 9.70	0.465	140.90 ± 9.88	139.18 ± 7.06	0.001	<0.001	<0.001
DBP (mm Hg)	70.97 ± 7.53	71.10 ± 7.41	0.759	67.20 ± 7.67	67.19 ± 4.07	0.978	<0.001	<0.001
Heart rate (beats/min)	68.74 ± 6.92	68.52 ± 6.76	0.565	66.39 ± 6.66	66.43 ± 6.58	0.919	<0.001	<0.001
Total cholesterol (mmol/L)	5.08 ± 0.62	5.03 ± 0.63	0.164	5.24 ± 0.76	4.64 ± 0.37	<0.001	<0.001	<0.001
Triglycerides (mmol/L)	1.50 ± 0.37	1.47 ± 0.37	0.159	1.52 ± 0.37	1.40 ± 0.32	<0.001	0.356	<0.001
HDL-c (mmol/L)	1.19 ± 0.21	1.20 ± 0.20	0.472	1.18 ± 0.23	1.24 ± 0.23	<0.001	0.436	<0.001
LDL-c (mmol/L)	3.20 ± 0.63	3.16 ± 0.65	0.223	3.38 ± 0.76	2.76 ± 0.55	<0.001	<0.001	<0.001
FPG (mmol/L)	5.46 ± 0.80	5.45 ± 0.75	0.809	5.49 ± 0.89	5.52 ± 1.01	0.597	0.543	0.140

*Data are expressed as the mean ± standard deviation or numbers with percentages. SBP, systolic blood pressure; DBP, diastolic blood pressure; TCHO, total cholesterol; TG, triglycerides; HDL-c, high-density lipoprotein cholesterol; LDL-c, low-density lipoprotein cholesterol; FPG, fasting plasma glucose*.

* *Baseline vs. end of follow-up in the placebo group*.

** *Baseline vs. end of follow-up in the rosuvastatin group*.

### Plasma Lipid Changes During Follow-Up

**Figure 1:**
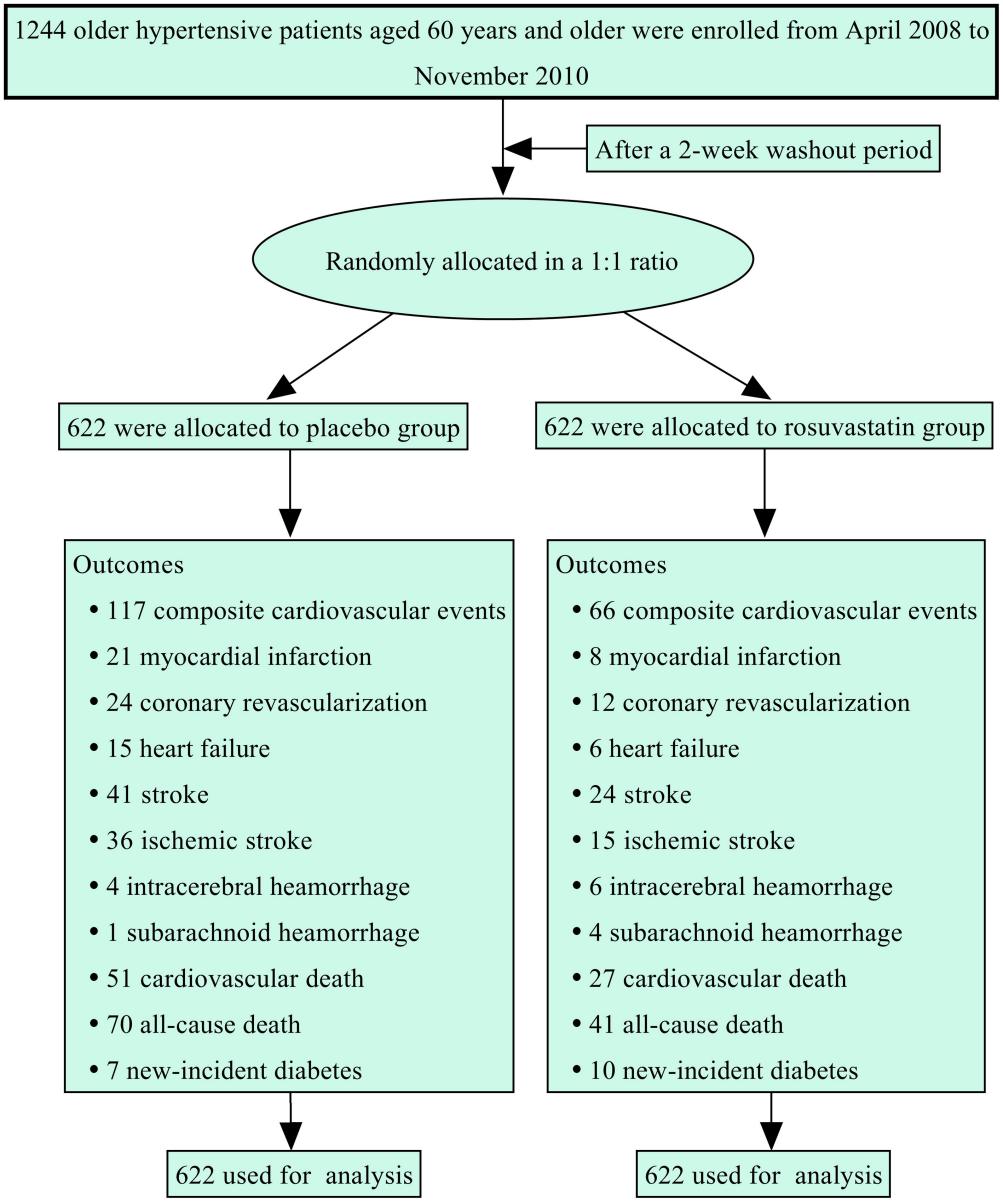
The protocol flowchart.

Figure 1 summarizes the protocol flowchart of this study. The average follow-up time was 83.5 (IQR: 80.0–86.0) months. The mean, SD, and CV in lipids during follow-up are shown in Table 2. There were significant differences in the trajectories of TCHO, TG, HDL-c, and LDL-c during the duration of the trial between the two groups after adjustment for age, sex, body mass index, and baseline lipid levels (*p* < 0.001, Supplementary Figure 1). The SDs for TCHO, TG, and LDL-c were significantly lower in the rosuvastatin group than the placebo group (all *p* < 0.001). The means and CVs for TCHO, TG, HDL-c, and LDL-c were significantly lower in the rosuvastatin group than the placebo group (all *p* < 0.05).

**Table 2:** Inter-visit plasma lipid variability during the follow-up period.

	**Placebo group**	**Rosuvastatin group**	***P-*value**
**TCHO**			
Mean (mmol/L)	5.28 ± 0.60	4.67 ± 0.49	<0.001
SD (mmol/L)	0.67 (0.50–0.85)	0.52 (0.40–0.68)	<0.001
CV (%)	12.96 (9.93–16.02)	11.06 (8.40–14.09)	<0.001
**TG**			
Mean (mmol/L)	1.51 ± 0.36	1.41 ± 0.32	<0.001
SD (mmol/L)	0.11 (0.08–0.14)	0.09 (0.07–0.13)	<0.001
CV (%)	7.21 (5.76–10.07)	6.75 (4.76–9.69)	<0.001
**HDL-c**			
Mean (mmol/L)	1.18 ± 0.21	1.24 ± 0.20	<0.001
SD (mmol/L)	0.13 (0.10–0.17)	0.13 (0.10–0.17)	0.654
CV (%)	11.43 (8.65–15.16)	10.83 (8.14–14.04)	0.001
**LDL-c**			
Mean (mmol/L)	3.41 ± 0.63	2.79 ± 0.50	<0.001
SD (mmol/L)	0.66 (0.47–0.83)	0.50 (0.38–0.64)	<0.001
CV (%)	19.88 (14.00–25.09)	18.06 (13.81–23.14)	0.004

*Data are expressed as the mean ± standard deviation or median interquartile range. TCHO indicates total cholesterol; TG, triglycerides; HDL-c, high-density lipoprotein cholesterol; LDL-c, low-density lipoprotein cholesterol; SD, standard deviation; CV, coefficient of variation*.

### Blood Pressure and FPG Changes During Follow-Up

The changes in BP and FPG are shown in Supplementary Figure 2 and Supplementary Table 1. The mean SBP was 139.52 ± 9.81 mm Hg in the placebo group and 137.87 ± 9.37 mm Hg in the rosuvastatin group during the follow-up. The differences in the trajectory and mean SBP were statistically significant between the placebo and rosuvastatin groups (*p* = 0.041 and 0.003, respectively). Differences were not found in the SDs and CVs for SBP, DBP, and FPG as well as the trajectory of DBP and FPG between the two groups.

### Primary Outcomes

**Figure 2:**
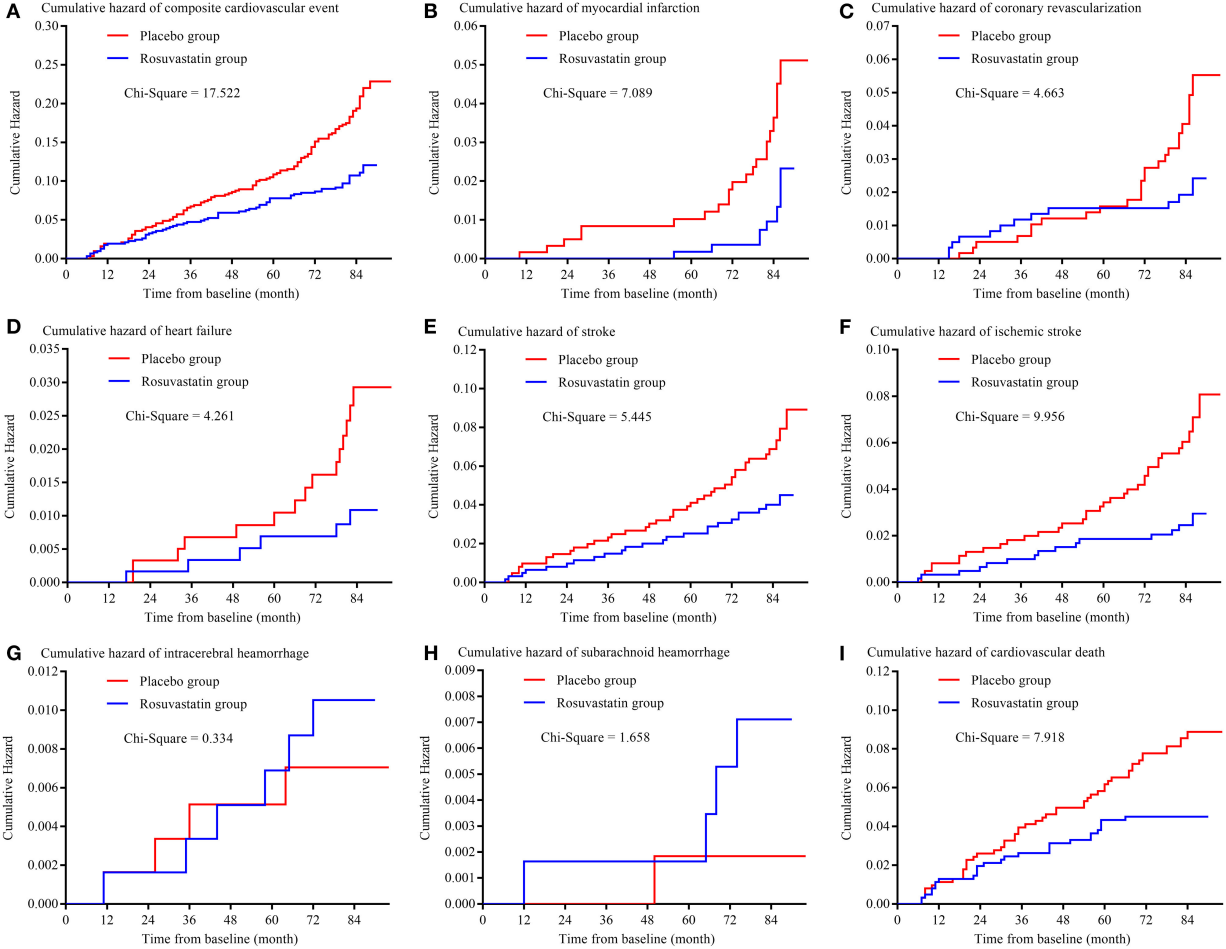
Kaplan-Meier curve of primary outcomes. **(A)** is the Kaplan-Meier curve of composite cardiovascular event; **(B)** is the Kaplan-Meier curve of myocardial infarction; **(C)** is the Kaplan-Meier curve of coronary revascularization; **(D)** is the Kaplan-Meier curve of heart failure; **(E)** is the Kaplan-Meier curve of stroke; **(F)** is the Kaplan-Meier curve of ischemic stroke; **(G)** is the Kaplan-Meier curve of intracerebral hemorrhage; **(H)** is the Kaplan-Meier curve of subarachnoid hemorrhage; **(I)** is the Kaplan-Meier curve of cardiovascular death.

Figure 2 and Table 3 presents the details of the risk of primary outcomes in the placebo and rosuvastatin groups. In unadjustment models, the risks of composite cardiovascular event, myocardial infarction, coronary revascularization, heart failure, total stroke, ischemic stroke, and cardiovascular death were significantly lower in the rosuvastatin group than the placebo group (all *p* < 0.05). Although the differences in the risks were decreased between the two groups, the risks of composite cardiovascular event, myocardial infarction, heart failure, total stroke, ischemic stroke, and cardiovascular death were significantly lower in the rosuvastatin group than the placebo group after adjustment for age, sex, smoking, alcohol consumption, education, baseline body mass index, mean and variability in SBP and DBP during the follow-up, and mean TCHO, TG, HDL-c, and LDL-c during the follow-up (all *p* < 0.05). The differences further diminished between the two groups after the CVs for TCHO, TG, HDL-c, and LDL-c were separately included as confounders.

**Table 3:** Cumulative hazards of outcomes in the rossuvastatin group compared with the placebo group.

	**Outcomes [*****n*** **(%)]**		**Model 1**		**Model 2**		**Model 3**	
	**Placebo group**	**Rosuvastatin group**	**HR (95% CI)**	***P*-value**	**HR (95% CI)**	***P*-value**	**HR (95% CI)**	***P*-value**
**Primary outcome**								
Composite cardiovascular event	117 (18.81)	65 (10.45)	0.523 (0.386 to 0.708)	<0.001	0.567 (0.389–0.827)	0.003	0.659 (0.436–0.998)	0.049
**Components**								
Myocardial infarction	21 (3.38)	8 (1.29)	0.348 (0.154–0.785)	0.011	0.401 (0.162–0.993)	0.046	0.402 (0.158–1.022)	0.056
Coronary revascularization	24 (3.86)	12 (1.93)	0.474 (0.237–0.949)	0.035	0.588 (0.264–1.308)	0.193	0.635 (0.279–1.442)	0.278
Heart failure	15 (2.41)	6 (0.96)	0.203 (0.065, 0.632)	0.006	0.247 (0.079–0.772)	0.011	0.277 (0.084–0.911)	0.035
Stroke	41 (6.59)	23 (3.70)	0.554 (0.334–0.916)	0.021	0.609 (0.401–0.925)	0.029	0.685 (0.450–1.043)	0.072
Ischemic stroke	36 (5.79)	15 (2.41)	0.392 (0.215–0.717)	0.002	0.433 (0.251–0.749)	0.014	0.526 (0.274–1.009)	0.056
Intracerebral hemorrhage	4 (0.64)	6 (0.96)	1.740 (0.509–5.944)	0.377	1.259 (0.293–5.420)	0.757	1.080 (0.608–1.917)	0.794
Subarachnoid hemorrhage	1 (0.16)	2 (0.32)	3.811 (0.426–34.096)	0.232	2.566 (0.203–32.420)	0.466	3.226 (0.220–47.341)	0.393
Cardiovascular death	51 (8.20)	28 (4.50)	0.518 (0.325–0.826)	0.006	0.492 (0.269–0.902)	0.022	0.659 (0.328–1.325)	0.242
**Secondary outcome**								
All-cause death	70 (11.25)	41 (6.59)	0.571 (0.388–0.839)	0.004	0.512 (0.313–0.838)	0.008	0.654 (0.427–1.002)	0.053
New-incident diabetes	7 (1.13)	10 (1.61)	1.375 (0.523–3.614)	0.518	1.014 (0.386–2.667)	0.724	1.230 (0.394–3.840)	0.905
**Primary outcome**								
Composite cardiovascular event	117 (18.81)	65 (10.45)	0.663 (0.442–0.995)	0.047	0.644 (0.429–0.966)	0.033	0.723 (0.481–1.089)	0.121
**Components**								
Myocardial infarction	21 (3.38)	8 (1.29)	0.473 (0.185–1.209)	0.118	0.437 (0.171–1.120)	0.085	0.474 (0.185–1.213)	0.119
Coronary revascularization	24 (3.86)	12 (1.93)	0.642 (0.284–1.453)	0.288	0.787 (0.337–1.841)	0.581	0.625 (0.277–1.411)	0.258
Heart failure	15 (2.41)	6 (0.96)	0.280 (0.086, 0.913)	0.035	0.254 (0.076–0.844)	0.025	0.259 (0.080–0.843)	0.025
Stroke	41 (6.59)	23 (3.70)	0.659 (0.431–1.007)	0.061	0.707 (0.461–1.084)	0.089	0.726 (0.477–1.105)	0.101
Ischemic stroke	36 (5.79)	15 (2.41)	0.525 (0.292–0.944)	0.023	0.552 (0.254–1.201)	0.134	0.621 (0.285–1.354)	0.231
Intracerebral hemorrhage	4 (0.64)	6 (0.96)	1.120 (0.246–5.099)	0.884	1.287 (0.292–5.675)	0.739	1.110 (0.250–4.933)	0.891
Subarachnoid hemorrhage	1 (0.16)	2 (0.32)	6.300 (0.748–53.061)	0.690	3.857 (0.243–61.272)	0.339	3.252 (0.222–47.589)	0.389
Cardiovascular death	51 (8.20)	28 (4.50)	0.620 (0.314–1.225)	0.169	0.565 (0.281–1.137)	0.110	0.580 (0.290–1.162)	0.124
**Secondary outcome**								
All-cause death	70 (11.25)	41 (6.59)	0.651 (0.428–0.990)	0.041	0.545 (0.316–0.939)	0.029	0.603 (0.350–1.037)	0.068
New-incident diabetes	7 (1.13)	10 (1.61)	1.081 (0.313–3.725)	0.902	1.072 (0.314–3.664)	0.912	1.017 (0.299–3.460)	0.979

*Model 1 adjustment for no confounders. Model 2 adjustment for age, sex, smoking, alcohol consumption, education, baseline body mass index, mean and variability in SBP and DBP during the follow-up, and mean TCHO, TG, HDL-c, and LDL-c during the follow-up. Model 3 adjustment for the confounders included in Model 2 followed by CV in TCHO during the follow-up. Model 4 adjustment for the confounders included in Model 2 followed by CV in TG during the follow-up. Model 5 adjustment for the confounders included in Model 2 followed by CV in HDL-c during the follow-up. Model 6 adjustment for the confounders included in Model 2 followed by CV in LDL-c during the follow-up. HR indicates hazard ratio; CI, confidence interval*.

### Secondary Outcomes

**Figure 3:**
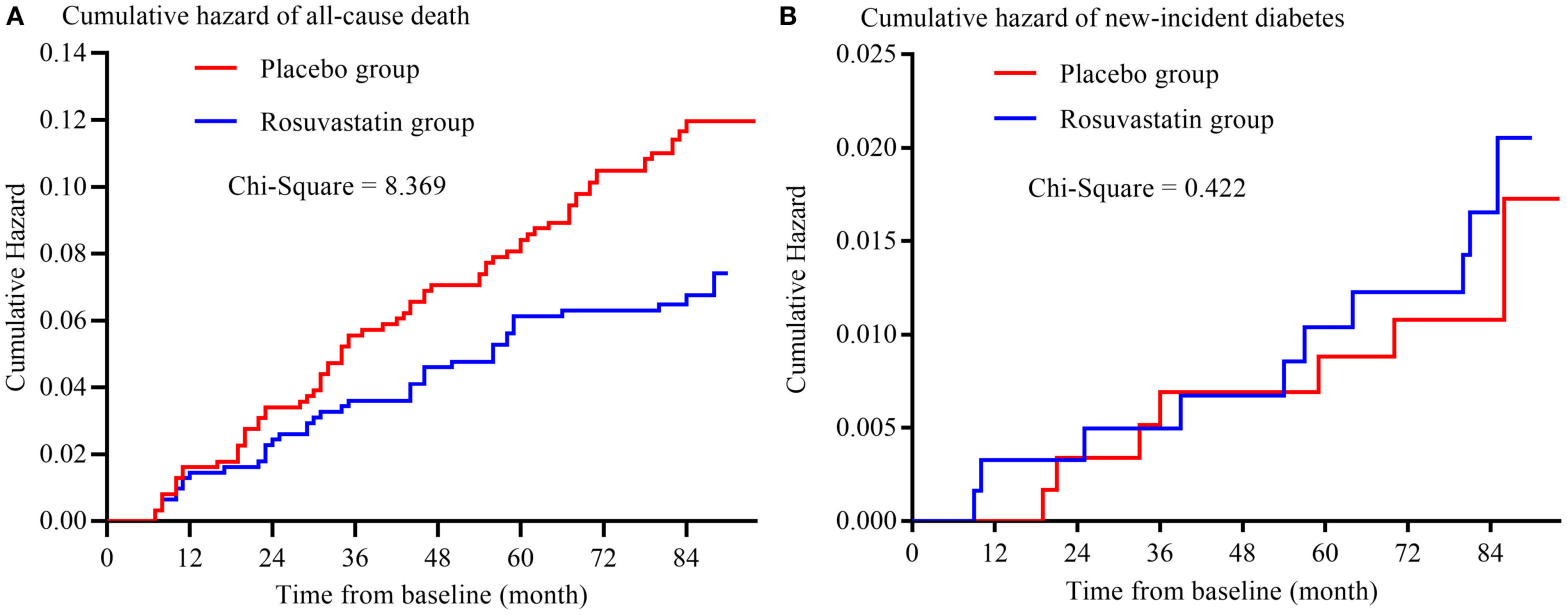
Kaplan-Meier curve of secondary outcomes. **(A)** is the Kaplan-Meier curve of all-cause death; **(B)** is the Kaplan-Meier curve of new-incident diabetes.

The risks of secondary outcomes are shown in Figure 3 and Table 3. There was no significant difference in the risk of new-incident diabetes between the placebo and rosuvastain groups. The risk of all-cause death was statistically lower in the rosuvastatin group than the placebo group (*p* = 0.004). Similar as the risks of primary outcomes, the difference in the risk of all-cause death decreased between two groups after adjustment for confounders and further diminished after the CVs for TCHO, TG, HDL-c, and LDL-c were separately included as confounders.

### Association Between the Risk of Outcomes and the Variability in Lipids

Table 4 shows the details of the association between the risk of outcomes and the variability in lipids during the follow-up period. After adjustment for age, sex, smoking, alcohol consumption, education, baseline body mass index, mean and variability in SBP and DBP during the follow-up, and mean TCHO, TG, HDL-c, and LDL-c during the follow-up, 1-SD of CVs for TCHO, TG, HDL-c, and LDL-c increment were significantly associated with the risks of composite cardiovascular event, myocardial infarction, heart failure, total stroke, ischemic stroke, cardiovascular death, and all-cause death, respectively (all *p* < 0.05). CVs for HDL-c, and LDL-c were significantly associated with the risk of coronary revascularization (all *p* < 0.05).

**Table 4:** Cumulative hazards of outcomes with each one-SD increment in the CVs for lipids during the follow-up period.

	**One-SD increase in CV TCHO**		**One-SD increase in CV TG**		**One-SD increase in CV HDL-c**		**One-SD increase in CV LDL-c**	
	**HR (95% CI)**	***P*-value**	**HR (95% CI)**	***P*-value**	**HR (95% CI)**	***P*-value**	**HR (95% CI)**	***P*-value**
**Primary outcome**								
Composite cardiovascular event	2.133 (1.850–2.460)	<0.001	1.689 (1.501–1.900)	<0.001	1.857 (1.635–2.109)	<0.001	2.381 (2.062–2.750)	<0.001
**Components**								
Myocardial infarction	1.231 (1.002–1.512)	0.017	1.473 (1.093–1.986)	0.011	1.436 (1.057–1.952)	0.021	1.663 (1.193–2.318)	0.003
Coronary revascularization	1.171 (0.935–1.467)	0.160	1.229 (0.925–1.634)	0.154	1.263 (1.008–1.583)	0.022	1.726 (1.343–2.218)	<0.001
Heart failure	1.632 (1.095–2.431)	0.016	1.485 (1.025–2.151)	0.036	1.788 (1.255–2.546)	0.001	1.733 (1.132–2.653)	0.011
Stroke	2.661 (2.130–3.326)	<0.001	1.709 (1.418–2.058)	<0.001	1.707 (1.398–2.085)	<0.001	2.395 (1.911–3.000)	<0.001
Ischemic stroke	3.036 (2.355–3.914)	<0.001	1.697 (1.371–2.100)	<0.001	1.769 (1.413–2.214)	<0.001	2.442 (1.890–3.155)	<0.001
Intracerebral hemorrhage	1.701 (0.847–3.413)	0.136	1.321 (0.628–2.778)	0.463	1.181 (0.669–2.086)	0.566	1.362 (0.674–2.755)	0.389
Subarachnoid hemorrhage	1.211 (0.452–3.247)	0.703	3.877 (0.680–22.105)	0.681	2.129 (0.888–5.103)	0.090	1.206 (0.471–3.087)	0.697
Cardiovascular death	2.200 (1.757–2.755)	<0.001	1.924 (1.593–2.323)	<0.001	1.879 (1.536–2.343)	<0.001	3.273 (2.535–4.227)	<0.001
**Secondary outcome**								
All-cause death	1.869 (1.552–2.250)	<0.001	1.818 (1.552–2.130)	<0.001	1.523 (1.269–1.826)	<0.001	2.370 (1.940–2.896)	<0.001
New-incident diabetes	1.313 (0.938–1.837)	0.1096	1.195 (0.695–2.053)	0.519	0.959 (0.477–1.930)	0.907	1.355 (0.690–2.660)	0.377

*Confounders included age, sex, smoking, alcohol consumption, education, baseline body mass index, mean and variability in SBP and DBP during the follow-up, and mean TCHO, TG, HDL-c, and LDL-c during the follow-up. SD indicates standard deviation; CV, coefficient of variation; TCHO, total cholesterol; TG, triglycerides; HDL-c, high-density lipoprotein cholesterol; LDL-c, low-density lipoprotein cholesterol; HR, hazard ratio; CI, confidence interval*.

## Discussion

Findings from this randomized clinical trial showed that: (1) not only the mean but also the variability in TCHO, TG, HDL-c, and LDL-c during the follow-up was significantly lower in the rosuvastatin group than the placebo group; (2) the risks of composite cardiovascular event, myocardial infarction, heart failure, total stroke, ischemic stroke, cardiovascular death, and all-cause death were significantly lower in the rosuvastatin group than the placebo group after adjustment for confounders including the means of TCHO, TG, HDL-c, and LDL-c during follow-up; (3) the differences in the risks of primary and secondary outcomes decreased or even vanished after the CVs for TCHO, TG, HDL-c, and LDL-c were separately included as confounders.

Statins are a class of medications that lower LDL-c and TCHO levels and elevating HDL-c levels in the bloodstream and exert protective effects against cardio- and cerebro-vascular diseases (12–17). The effects of statins have been shown to be dose-dependent (12, 34, 35). In this study, the levels of TCHO, TG, and LDL-c were markedly lower, and the level of HDL-c was higher, in the rosuvastatin group than the placebo group. The risks of composite cardiovascular event, myocardial infarction, heart failure, ischemic stroke, cardiovascular death, and all-cause death were significantly lower in the rosuvastatin group than the placebo group. Statins have been shown to exert protective effects against cardio- and cerebro-vascular diseases (25, 36–38). The results of the HOPE-3 (Heart Outcomes Prevention Evaluation) trial have revealed that low-dose rosuvastatin decreases cardiovascular diseases in participants with two or more healthful lifestyle factors (HR: 0.74 with 95% CI: 0.62–0.90) and in participants with fewer than two factors (HR: 0.79 with 95% CI: 0.61–1.01) (25). Thus, rosuvastatin, even with low-dose, effectively modulates plasma lipids and decreases the risk of cardiovascular events in older patients with antihypertensive treatment.

Furthermore, we also found that rosuvastatin, which was administrated 10 mg once daily, could significantly decreased the variability in plasma lipids including TCHO, TG, HDL-c, and LDL-c. After the intra-individual SD was corrected for intraindividual mean lipid levels over the same measurement period, the CVs for TCHO, TG, HDL-c, and LDL-c were statistically lower in the rosuvastatin group than the placebo group. We further separately included the CVs for TCHO, TG, HDL-c, and LDL-c as confounders in different Cox hazard models. The results, as expected, showed that the differences in the risks of primary and secondary outcomes between the placebo and rosuvastatin groups were markedly decreased in the CV for TCHO model, CV for TG model, CV for HDL-c model, and CV for LDL-c model.

A few studies have suggested that the variability in LDL-c is associated with cardiovascular disease (10, 11, 39). Our results were in good agreement with findings from previous studies. Moreover, unexpectedly, the excessive variability in TCHO, TG, and HDL-c was independently and closely associated with a higher risk of composite cardiovascular event, myocardial infarction, heart failure, total stroke, ischemic stroke, cardiovascular death, and all-cause death in this study. The association remained after adjustment for confounders including the means of TCHO, TG, and HDL-c during follow-up. It indicates that the effects of variability in TCHO, TG, and HDL-c are therefore as important as the variability in LDL-c in cardiovascular events, although their importance might be inconsistent.

In this study, we did not find significant differences in the risks of intracerebral hemorrhage, subarachnoid hemorrhage, and newly incident diabetes mellitus between the placebo and rosuvastatin groups, although the cumulative incidence rates of intracerebral hemorrhage, subarachnoid hemorrhage, and newly incident diabetes mellitus were lower in the placebo group than the rosuvastatin group. Low-dose rosuvastatin administration may be an important cause of these results. Low-dose rosuvastatin has been shown to cause fewer potential side-effects than high dose rosuvastatin (37, 40). In addition, some beneficial pleiotropic effects of rosuvastatin such as anti-inflammation and anti-oxidation are not strictly dose dependent (37). On the other hand, we did not found the association of the variability in lipids with the risks of intracerebral hemorrhage, subarachnoid hemorrhage, and newly incident diabetes mellitus. These findings might have resulted primarily from the low incidence of these events during follow-up.

## Strengths and Limitations

The major strengths of this study were its long-term follow-up period and adequate sample size. The participants were followed up for an average of 83.5 (IQR: 80.0–86.0) months. Another strength of this study was its randomized, double-blind, placebo-controlled clinical trial design. The long duration of follow-up, adequate sample size, and study design minimized the bias of the results as much as possible.

The main limitation of this study was that different types and doses of statins were not considered. The effects of different types and doses of statins on the variability in lipids, especially in TCHO, TG, and HDL-c, were not fully elucidated, although the variability in LDL-c was found to be consistent among the different statins (including rosuvastatin, simvastatin, and atorvastatin) and doses (41). Second, genetic polymorphisms were not included as a factor in this study. Genetic polymorphisms have been found to often be associated with individual variability in LDL-c (42, 43). Slimani et al. have reported that the proprotein convertase subtilisin kexine 9 and apolipoprotein E genes are involved in LDL-c response variability (42). Third, the antihypertensive treatments maybe affect the lipids variability and outcomes and induce bias in the results. However, the randomized design of the trial reduced this affect. Moreover, this affect could be further limited because that mean and variability in SBP and DBP during the follow-up period were included as confounders in models. In addition, it is uncertain whether our findings are applicable to other ethnic groups, because the ethnicity of participants in this study was mainly Han nationality in the Shandong area. In conclusion, our results indicated that excessive variability in lipids included TCHO, TG, HDL-c, and LDL-c were independently associated with higher risks of cardiovascular mortality and morbidity.

In conclusion, our results indicated that rosuvastatin efficiently decreased the risk of cardiovascular mortality and morbidity in older patients with antihypertensive treatment. The mechanism underlying this effect might be that rosuvastatin significantly attenuated the variability in lipids besides modulated the levels of plasma lipids. In the management of hypertensive patients, controlling the level and variability of lipid profiles is as important as antihypertensive treatment to reduce the cardiovascular morbidity and mortality. However, multiracial, multinational, and larger sample size clinical trials incorporating other types and doses of statins are needed to validate our results.

## Data Availability Statement

The original contributions presented in the study are included in the article/Supplementary Material, further inquiries can be directed to the corresponding author/s.

## Ethics Statement

The studies involving human participants were reviewed and approved by Research Ethics Committee of the Shandong Academy of Medical Sciences. The patients/participants provided their written informed consent to participate in this study.

## Author Contributions

YD and XL conducted the analyses, wrote the methods, results, and discussion sections. YZ developed the introduction section and collected the data. QC revised earlier versions of the manuscript. HZ co-conducted the analyses, results, and revised earlier versions of the manuscript. YG co-designed the study and developed the discussion sections. ZL designed and initiated the study, conducted the analyses, developed the introduction and discussion sections, and revised final versions of the manuscript. All authors contributed to the article and approved the submitted version.

## Conflict of Interest

The authors declare that the research was conducted in the absence of any commercial or financial relationships that could be construed as a potential conflict of interest.
