# Supplementary material for: Attenuating the Variability of Lipids Is Beneficial for the Hypertension Management to Reduce the Cardiovascular Morbidity and Mortality in Older Adults
Source: Front Cardiovasc Med. 2021 Jun 17;8:692773. doi: 10.3389/fcvm.2021.692773 (PMC8245783; doi:10.3389/fcvm.2021.692773)
Supplement: Supplementary file 1 [file Data_Sheet_1.pdf]

Supplementary Materials:

Attenuating the variability of lipids is beneficial for the hypertension management to reduce the cardiovascular morbidity and mortality in older adults

Short title: Lipids variability and hypertension management

Yuanli Dong[1]†, Xukui Liu[2]†, Yingxin Zhao[2,3], Qiang Chai[2,3], Hua Zhang[2,3], Yumei Gao[4*], Zhendong Liu[2,3*]

Supplementary Table 1. Inter-visit blood pressure and fasting plasma glucose variability during the follow-up period.

Supplementary Figure 1. Trajectory of blood pressure and fasting plasma glucose during the trial.

Supplementary Figure 2. Trajectory of blood pressure and fasting plasma glucose during the trial.

Supplementary Table 1. Inter-visit blood pressure and fasting plasma glucose variability during the follow-up period

	**Placebo group**	**Rosuvastatin group**	***P* value**
Systolic blood pressure			
Mean (mm Hg)	139.52±9.81	137.87±9.37	.003
SD (mm Hg)	12.90±2.28	12.97±2.40	.578
CV (%)	9.26±1.64	9.42±1.66	.097
Diastolic blood pressure			
Mean (mm Hg)	67.11±7.42	67.00±7.46	.802
SD (mm Hg)	7.99±0.92	7.89±0.89	.068
CV (%)	12.07±2.02	11.94±1.97	.249
Fasting plasma glucose			
Mean (mmol/L)	5.43±0.53	5.46±0.51	.299
SD (mmol/L)	0.72±0.29	0.71±0.27	.568
CV (%)	13.34±5.24	13.09±5.00	.393

Data are expressed as the mean±standard deviation. SD indicates standard deviation; CV, coefficient of variation.

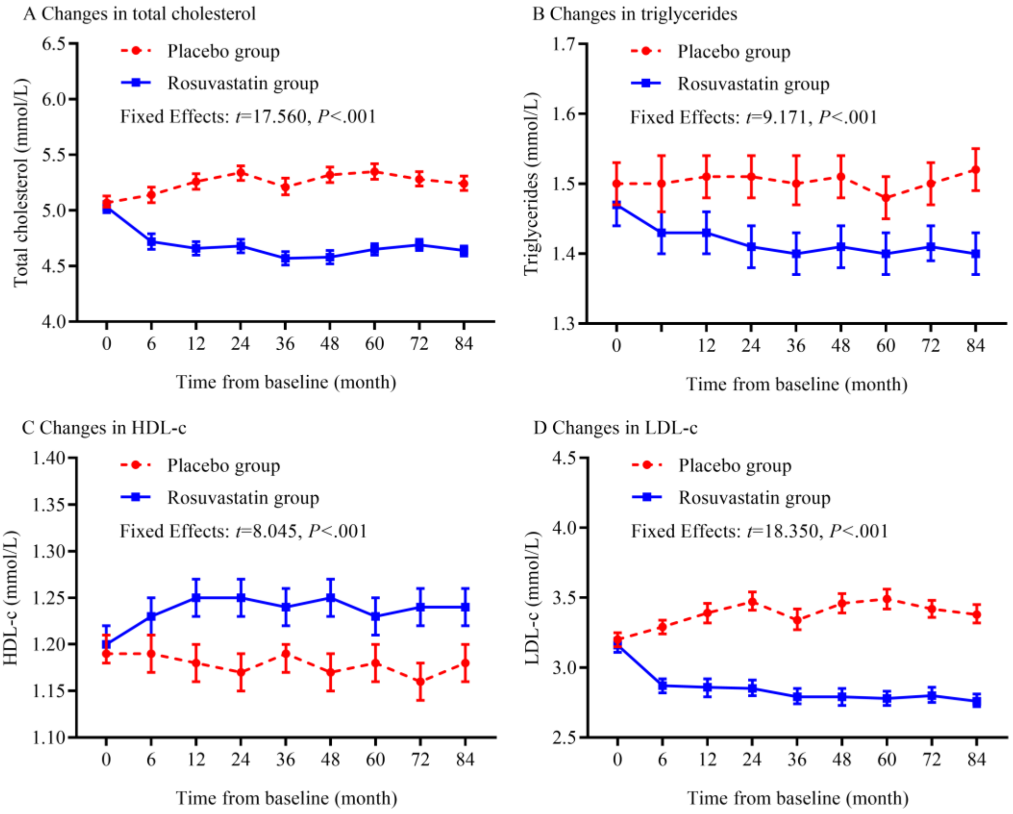

Supplementary Figure 1. Trajectory of plasma lipids during the trial. A is the trajectory of total cholesterol; B is the trajectory of triglycerides; C is the trajectory of high-density lipoprotein cholesterol; and D is the trajectory of low-density lipoprotein cholesterol. Results are means with 95% confidence interval (error bars). TCHO, total cholesterol; TG, triglycerides; HDL-c, high-density lipoprotein cholesterol; LDL-c, low-density lipoprotein cholesterol. Results are means with standard error (error bars).

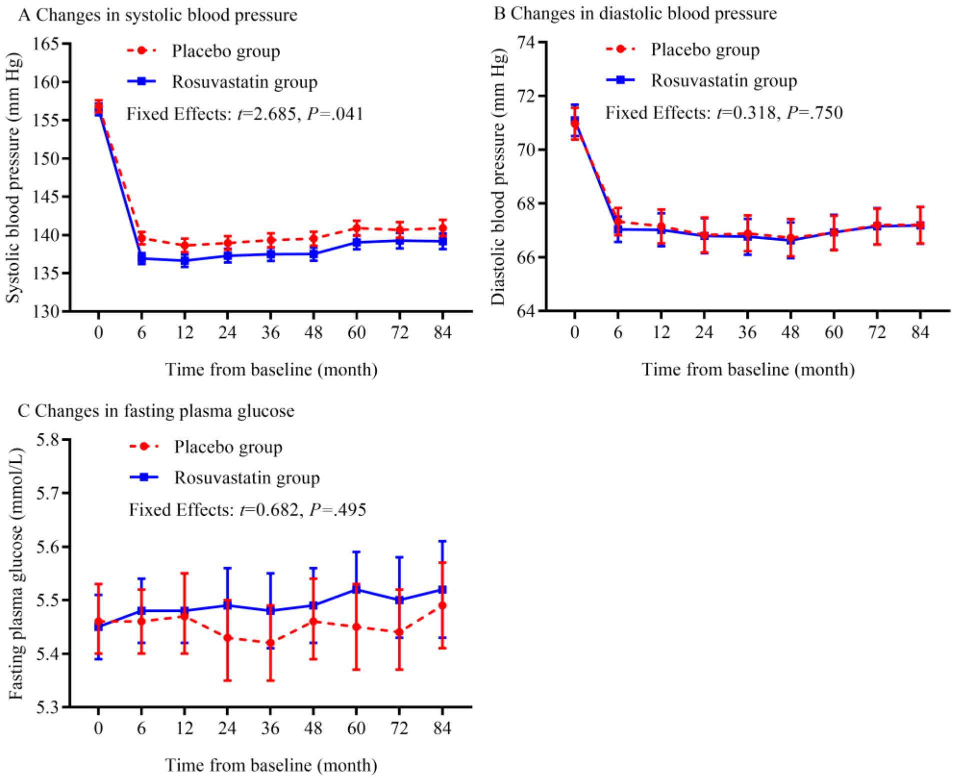

Supplementary Figure 2. Trajectory of blood pressure and fasting plasma glucose during the trial.

A is the trajectory of systolic blood pressure; B is the trajectory of diastolic blood pressure; and C is the trajectory of fasting plasma glucose. Results are means with standard error (error bars).
